# Inhibition of PC4 radiosensitizes non‐small cell lung cancer by transcriptionally suppressing XLF

**DOI:** 10.1002/cam4.1332

**Published:** 2018-03-09

**Authors:** Tian Zhang, Xiaojie Liu, Xiuli Chen, Jing Wang, Yuwen Wang, Dong Qian, Qingsong Pang, Ping Wang

**Affiliations:** ^1^ Department of Radiotherapy National Clinical Research Center for Cancer Tianjin's Clinical Research Center for Cancer Key Laboratory of Cancer Prevention and Therapy Tianjin Medical University Cancer Institute and Hospital Tianjin China; ^2^ Department of Radiotherapy Tianjin Hospital Tianjin China

**Keywords:** Non‐small cell lung cancer, positive cofactor 4, radiosensitivity, transcriptional regulation, XRCC4‐like factor

## Abstract

Positive cofactor 4 (PC4) participates in DNA damage repair and involved in nonhomologous end joining (NHEJ). Our previous results demonstrated that knockdown of PC4 downregulated the expression of XRCC4‐like factor (XLF) in esophageal squamous cell carcinoma. However, the mechanism how PC4 regulates the expression of XLF remains unclear. Here, we found that knockdown of PC4 increased radiosensitivity of non‐small cell lung cancer (NSCLC) both in vivo and in vitro. Furthermore, we found that PC4 knockdown downregulated the expression of XLF, whereas recovering XLF expression restored radioresistance in the PC4‐knockdown NSCLC cells. In addition, PC4 knockdown inhibited XLF expression by transcriptionally suppressing of XLF. Moreover, PC4 expression correlated with radiosensitivity and was an independent prognostic factor of progression‐free survival (PFS) in patients with NSCLC. These findings suggest that PC4 could be used as a promising therapeutic target for NSCLC.

## Introduction

Lung cancer is the most frequent cause of mortalities from cancer worldwide, and non‐small cell lung cancer (NSCLC) is the most common type of this malignancy 1. Radiotherapy plays an important role in the management of patients with NSCLC, particular those at advanced stages. However, the response to radiation treatment is variable and molecular markers that predict radiosensitivity are urgently required.

DNA double‐strand break (DSB) is regarded as the most lethal ionizing radiation (IR)‐related DNA lesions 2. Positive cofactor 4 (PC4) was first reported as transcriptional coactivators in vitro 3, and involved in varieties of processes including DNA damage repair and transcription. Previous study demonstrated that PC4 was recruited to DSB by microirradiation, which may be related to DNA repair 4, 5. Batta K et al. 6 found that PC4 promotes double‐stranded DNA ligation activity with X‐ray repair cross‐complementing protein 4 (XRCC4)–ligase IV complex, suggesting it may be involved in DSB repair, which is important for cancer radiosensitivity.

Our previous findings showed that inhibition of PC4 suppresses nonhomologous end joining (NHEJ) by regulating XLF in esophageal squamous cell carcinoma (ESCC) cells 7. However, the mechanism of PC4 regulating the expression of XLF remains unclear. The significance of PC4 on prognosis and therapeutic response in NSCLC has not been fully understood.

In our study, we report that PC4 is high expressed in NSCLC cells and tissues for the first time. Knockdown of PC4 increased the radiosensitivity of NSCLC by transcriptionally suppressing XLF. Furthermore, PC4 significantly correlated with NSCLC sensitivity to chemoradiotherapy and was an independent prognostic factor for poor PFS in patients with NSCLC.

## Materials and Methods

### Cell lines

NSCLC cell lines A549, H1975, H460 and PC‐9 and normal bronchial epithelial cells Beas‐2B were obtained from DSMZ (Germany). Beas‐2B was maintained in BEBM, A549, H1975, H460, and PC‐9 were maintained in RPMI‐1640 medium containing 10% fetal bovine serum, 1% penicillin, and 1% streptomycin. All cells were cultured at 37°C in 5% CO_2_. A549 and PC‐9 were characterized by Genetic Testing Biotechnology Corporation (Suzhou, China) using short tandem repeat (STR) markers (Data S1 and S2). All cells were mycoplasma tested before experiments every three months. An X‐ray machine (Precision X‐Ray Inc., USA) was used for radiation with 2.3 Gy/min dose rate.

### Colony formation assay

An increasing number of A549 and PC‐9 NSCLC cells were seeded in six‐well plates in triplicate. After attached to the culture flask, cells were exposed to the indicated doses of radiation using 6 MV X‐rays from linear accelerator at a dose rate of 2.3 Gy/min. Cells were incubated at 37°C of 10–14 days after irradiation. Colonies were fixed with 100% methanol and then stained with 1% crystal violet. Colonies consisting >50 cells were scored. SF = 1−(1−e^−D/D0^)^N^ was used to calculate surviving fraction.

### Construction of the recombinant lentiviral vector

See in Data S4.

### Western Blot assay

Whole‐cell proteins were extracted using RIPA buffer (Cell Signaling Technology, Danvers, MA, USA) containing proteinase inhibitor cocktail (Sigma, St. Louis, MO, USA). Protein (30 *μ*g) was subjected to 12% SDS‐PAGE gels and transferred onto PVDF membrane. The membrane was blocked with 5% nonfat milk for 1 h at room temperature (RT), then incubated with primary antibodies overnight at 4°C and secondary antibodies for 1 h at RT, and visualized by exposure apparatus (Biorad, Hercules, California, USA) after adding ECL (Millipore, Massachusetts, USA). PC4, XLF, DNA ligase IV, and XRCC4 antibodies were purchased from Abcam (Cambridge, MA, USA). *β*‐actin antibody was purchased from Cell Signaling Technology. Molecular weight of protein was shown in Data S3.

### Reverse transcription‐PCR

Reverse transcriptase‐PCR was used to examine PC4 and XLF mRNA expression. After extracted using TRIZOL reagent (Invitrogen, Carlsbad, CA, USA), total RNA was used for cDNA synthesis with Moloney murine leukemia virus reverse transcriptase (Promega, Madison, WI, USA). Amplification consisted of 30 cycles 7. The sequences of primers are listed in Table S1.

### Immunofluorescence

NSCLC cells were seeded into 24‐well culture plates with a glass coverslip over each well. The cells were fixed in 4% paraformaldehyde and then permeabilized with 1% Triton‐100. Subsequently, the cells were blocked with 10% goat serum for 30 min at RT. Cells were incubated with *γ*‐H2AX and XLF primary antibodies (1:100) and then incubated with secondary antibodies (1:100) for 1 hour at RT. Cells were then incubated with DAPI to stain the nuclei. Images were captured with fluorescence microscope (Olympus, Japan). The numbers of *γ*‐H2AX foci (red) and XLF foci (green) were counted in the field of vision by Image J software. XLF and *γ*‐H2AX antibodies were purchased from Abcam.

### Flow cytometric analysis of apoptosis

The percentage of apoptosis was tested using Annexin‐V fluorescein isothiocyanate (FITC) and propidium iodide (PI) apoptosis detection kit (BD Biosciences, Bedford, MA, USA) according to the protocol. Each sample was then subjected to analyses by flow cytometer (BD Biosciences, USA).

### Luciferase report assay

See in Data S4.

### Coimmunoprecipitation (Co‐IP)

See in Data S4.

### ChIP assays

See in Data S4.

### In vivo assay

4–6‐week‐old female athymic nude mice were used in xenograft experiments. Control PC‐9 cells and PC‐9 shPC4 cells were injected on the rear leg subcutaneously. The mice were randomized into four groups: Mock, shPC4, Mock + IR, and shPC4 + IR when tumors grew to 200 mm^3^. A total dose of 6 Gy (2 Gy/F every other day) was irradiated locally. Tumor volume was calculated using this formula: length × width^2^ /2.

### Patients and tissue specimens

Ninety‐one patients with NSCLC from 2009 to 2012 at Tianjin Medical University Cancer Institute and Hospital were enrolled in this study: 1. underwent surgery; 2. underwent adjuvant chemotherapy or chemoradiotherapy; 3. had pathological diagnosis and follow‐up data. The clinicopathological characteristics of patients were listed in Table 1.

**Table 1:** Characteristics of the 91 patients with NSCLC and according to PC4 nuclear expression determined in IHC

Characteristics	PC4		*P* value
	Low expression, N	High expression, N	
Gender			
Male	40	31	0.615
Female	10	10	
Age			
<58	24	20	0.941
≥58	26	21	
Pathological stage			
I‐II	28	21	0.649
III	22	20	
Pathological lymph node metastasis(pN)			
N0‐1	31	23	0.568
N2	19	18	
Histological type			
Squamous cell carcinoma	33	29	0.888
Adenocarcinoma	14	10	
Not specified	3	2	
Histological grade			
Well, moderately	32	30	0.350
Poorly	18	11	
Neoadjuvant chemotherapy			
Yes	34	30	0.591
No	16	11	
Adjuvant chemotherapy			
Yes	28	28	0.230
No	22	13	
Adjuvant radiotherapy			
yes	6	7	0.491
no	44	34	
Effect of neoadjuvant chemotherapy			
No‐effective group(SD + PD)	8	15	0.028*
Effective group(CR + PR)	26	15	

SD, stable disease; PD, progressive disease; CR, complete response; PR, partial response.

*P <* *0.05

### Tissue microarray and Immunohistochemistry (IHC)

Triplicate 0.6‐mm‐diameter cylinders were made using tissue array instrument. IHC was performed as described previously 8.

### Statistical analysis

Data of cell line experiments are analyzed by Student's t‐test. The associations between two characteristics were assessed by the Chi‐squared test. PFS and OS were analyzed with the Kaplan–Meier analysis. Multivariate analysis was conducted using Cox regression to identify independent prognostic factors. Statistical analysis was performed with the SPSS 21.0 software (IBM, Armonk, NY, USA). *P *<* *0.05 was considered statistically significant.

## Results

### PC4 levels modulate NSCLC cell radiosensitivity

**Figure 1:**
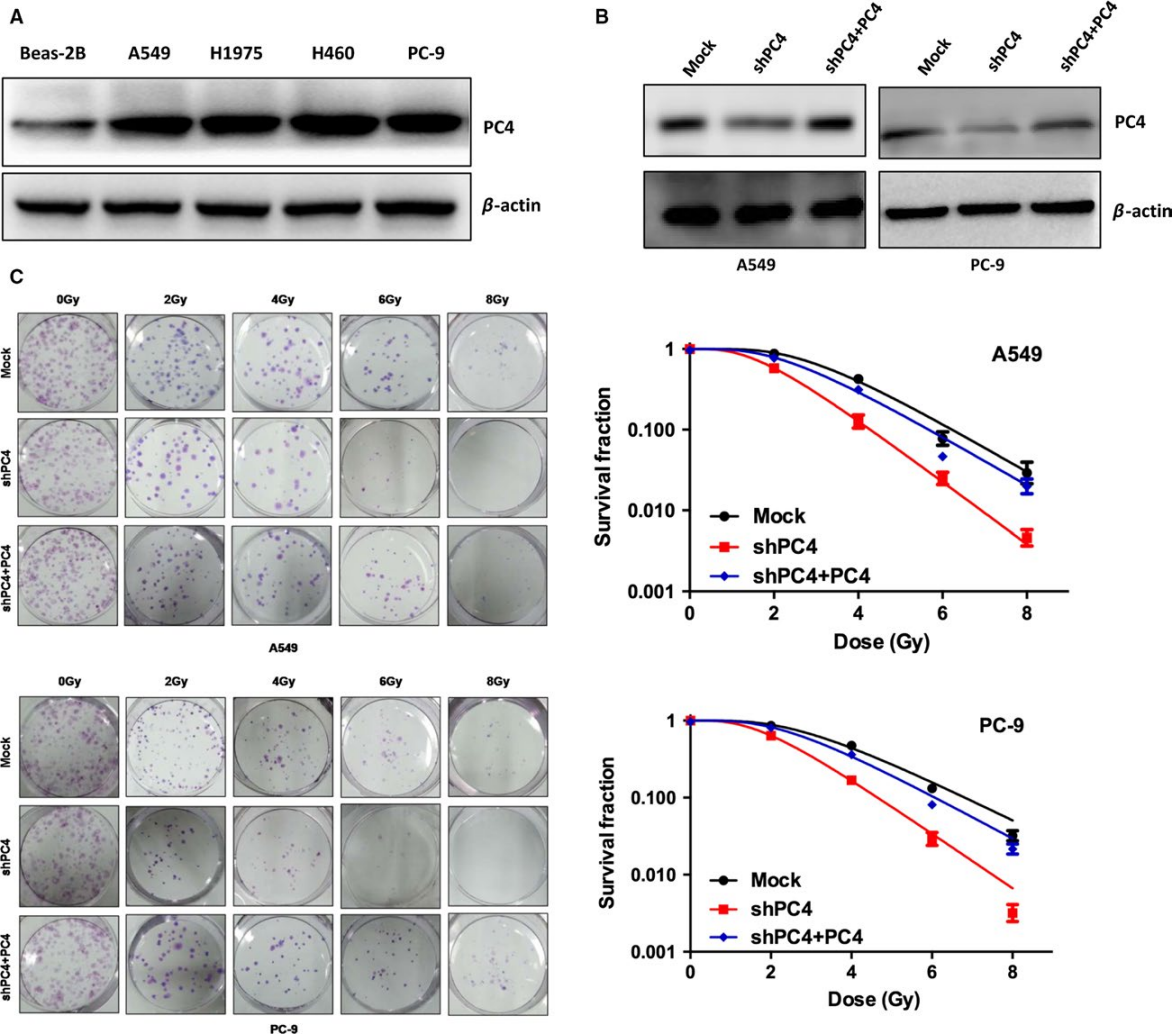
PC4 levels modulate NSCLC cell radiosensitivity in vitro. (A) Western blot analysis showed that PC4 levels in four NSCLC cell lines (A549, H1975, H460, and PC‐9) were higher than that in a normal bronchial epithelial cell line (Beas‐2B). (B) ShPC4 was introduced into two NSCLC cell lines (A549 and PC‐9) for stable knockdown of PC4 through recombinant lentiviral infection. Then, pCDH‐PC4 lentiviral particles were transduced into the above PC4‐silenced NSCLC cells (shPC4 + PC4) to replenish PC4 expression. PC4 levels were examined by Western blot. (C) Responses of NSCLC cells with different PC4 levels to IR were examined by colony formation assay. Mock, nonsilencing scramble RNA sequence control; shPC4, shRNA‐targeting PC4 mRNA. Data represent the mean ± S.E. of three individual experiments with triplicates.

All four NSCLC cell lines showed higher protein levels of PC4 than bronchial epithelial cell line Beas‐2B (Fig. 1A). PC4 was then knocked down by lentiviral infection (shPC4) in A549 and PC‐9 cells, which showed a good silencing effect (Fig. 1B). PC4‐knockdown NSCLC cells showed lower colony formation capacity than control cells after irradiation (Fig. 1C). To ensure that PC4 modulates radiosensitivity of NSCLC cells, PC4 level was replenished in PC4‐knockdown A549 and PC‐9 cells (Fig. 1B). The results demonstrated that replenishing the expression of PC4 enhanced the survival capacity in PC4‐silenced cell lines substantially (Fig. 1C). These data suggested that high expression of PC4 results in radioresistance in NSCLC cells.

### Knockdown of PC4 increases IR‐induced apoptosis

**Figure 2:**
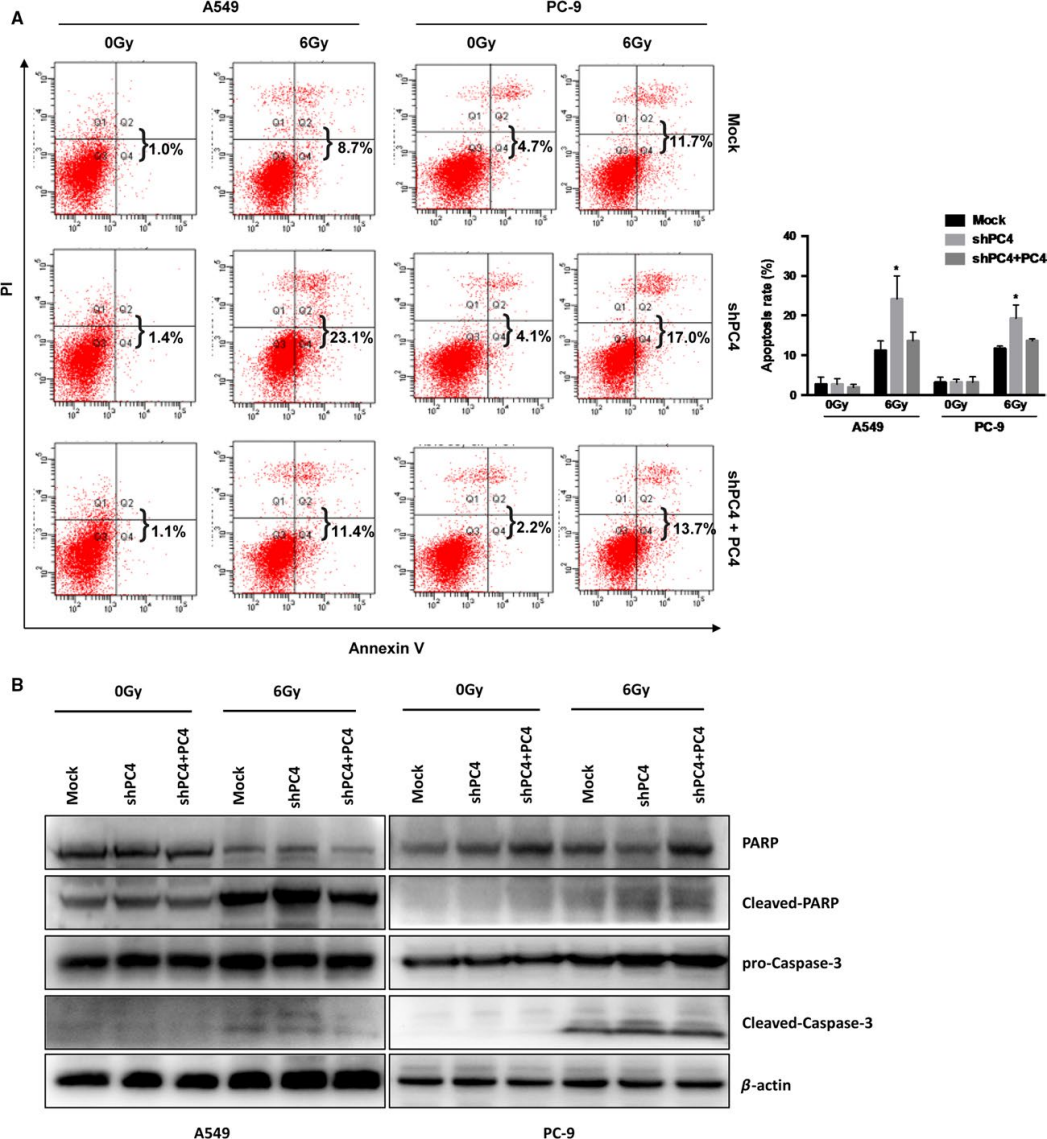
Silencing of PC4 promotes IR‐induced apoptosis. (A) Silencing of PC4 increased the percentage of apoptotic cells in both A549 and PC‐9 cell lines after 24 h exposure to 6 Gy IR. The IR‐induced cell apoptotic death events were monitored by annexin V/propidium iodide staining and flow cytometry (*P <* *0.05). (B) Silencing of PC4 increased the levels of cleaved PARP and cleaved caspase‐3 in both A549 and PC‐9 cells that were exposed to 6 Gy IR.

To examine the mechanism that PC4 knockdown‐induced radiosensitivity, flow cytometry was used to test the apoptosis rate in A549 and PC‐9 cells. The results demonstrated that PC4 knockdown did not increase apoptosis in normal condition. However, when the cells were treated with IR, it significantly increased the apoptotic rate (Fig. 2A, Table S2). After the cells were treated with IR, the expression of cleaved Caspase‐3 and cleaved PARP was increased in PC4‐knockdown cells (Fig. 2B). Taken together, low expression of PC4 increased the radiosensitivity of NSCLC cells by increasing apoptosis.

### Knockdown of PC4 increases the radiosensitivity of NSCLC in vivo

**Figure 3:**
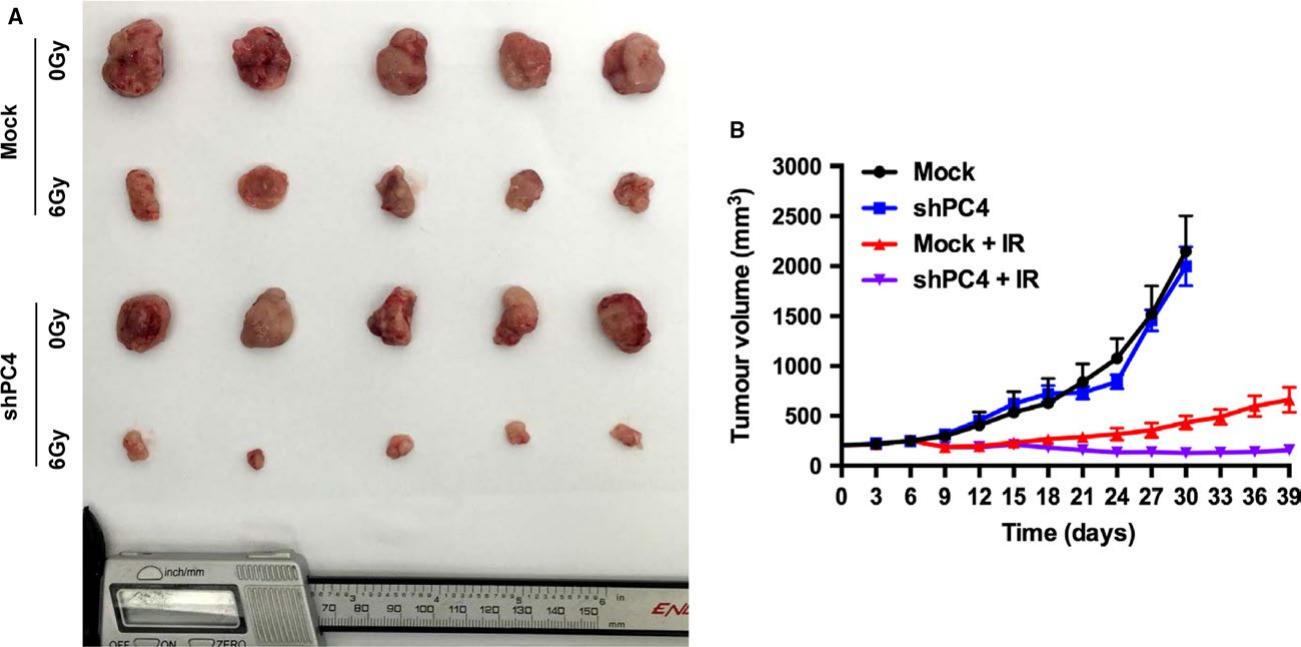
Inhibition of PC4 enhances the therapeutic effect of IR on NSCLC cell xenografts. PC‐9‐shPC4 and control PC‐9 cells (6 × 10^6^) were injected into the lower limb of female athymic nude mice. When the volume of a tumor reached 200 mm^3^, mice in the treatment groups were treated with 6 Gy IR. The xenograft tumors were removed on the 30th day in Mock and shPC4 groups, and on the 39th day on Mock + IR and shPC4 + IR groups. (A) Representative images showing xenografted tumors in null mice derived from PC‐9‐Mock and PC‐9‐shPC4 cells. (B) Tumor volumes of xenografts were measured by calipers every 3 days for 30–39 days. PC4 knockdown did not affect the growth of tumors in nontreatment groups (Mock and shPC4). Mean tumor volumes in Mock and shPC4 groups were 2149.7 ± 356.4 and 1999.7 ± 441.0 mm^3^, respectively (*n *= 5, *P *=* *0.73, Student's t‐test). After treatment with 6 Gy IR (Mock + IR and shPC4 + IR groups), the mean tumor volume in the shRNA group was 116.0 ± 45.0 mm^3^, which was significantly smaller than 660.2 ± 282.1 mm^3^ in the Mock group (*n *= 5, *P *=* *0.022, Student's t‐test). Values represent the mean tumor volume ± SE.

We then investigated whether PC4 knockdown affects the NSCLC radiosensitivity in vivo. Control PC‐9 cells and PC‐9‐shPC4 cells were injected into female athymic nude mice. The xenografted mice were treated with IR when tumors reached at least 200 mm^3^. We found that after treated with IR, tumors developed more slowly in mice bearing the PC‐9‐shPC4 xenograft than control group (Fig. 3A and B). These results confirmed that PC4 increases the radiosensitivity of NSCLC in vivo.

### Silencing of PC4 inhibits XLF through transcriptionally suppressing XLF

**Figure 4:**
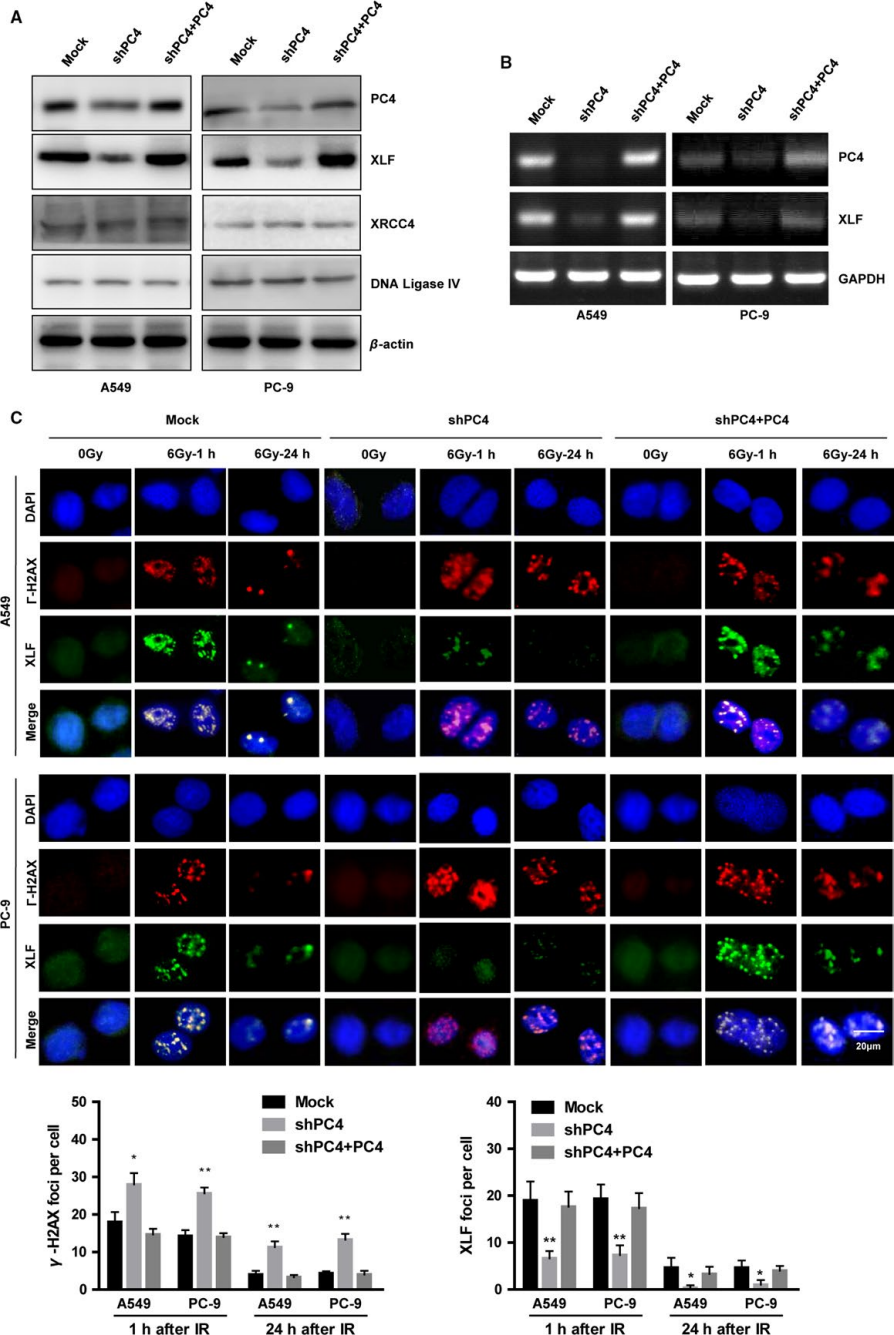
Silencing of PC4 inhibits the recruitment of XLF to DSB repair foci. (A) Inhibition of PC4 downregulated the protein expression of XLF. However, PC4 knockdown did not alter the expression of XRCC4 or DNA ligase IV. *β*‐actin was used as a loading control. (B) Silencing of PC4 downregulated the mRNA expression of XLF. GAPDH was used as a loading control. (C) Cells were subjected to IR (6 Gy). After 1 or 24 h, the cells were fixed for immunofluorescence (*γ*‐H2AX, red; XLF, green). Γ‐H2AX foci were used as a measure of DSBs. Merged spots (yellow) show colocalization of XLF and *γ*‐H2AX foci at DSBs (upper panel). Quantification of the average numbers of IR‐induced *γ*‐H2AX foci per cell (1 or 24 h after IR, lower panel). Data were quantified by multiple counts and plotted. Data represent mean values with S.E. (*P <* *0.05, **P <* *0.01, Student's t‐test).

NHEJ is a major pathway in repairing IR‐induced DSB. We found that inhibition of PC4 expression had no effect on the expression of XRCC4 or DNA ligase IV. However, XLF was downregulated by silencing PC4 at both protein and mRNA levels in A549 and PC‐9 cells (Fig. 4A and B). XLF foci colocalization with *γ*‐H2AX occurred in A549 and PC‐9 cells as early as 1 h treated with 6 Gy IR. Furthermore, XLF foci formation was reduced in A549 and PC‐9 cells by knockdown of PC4. In contrast, after 6 Gy IR treatment, the DSBs detected by *γ*‐H2AX were greater in PC4‐knockdown cells (Fig. 4C).

**Figure 5:**
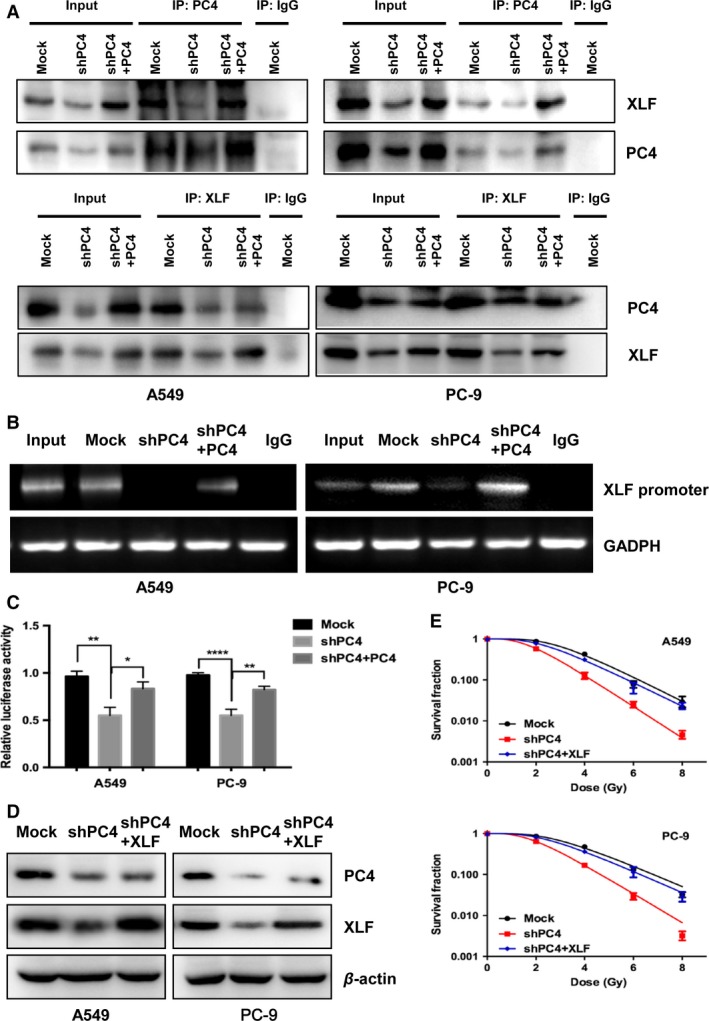
PC4 knockdown regulates XLF expression by transcriptionally suppressing XLF in NSCLC cells. (A) A549 and PC‐9 cells were assayed for protein expression levels by immunoprecipitation (IP) as described in the Materials and Methods. (B) Chromosome immunoprecipitation to measure the effects of PC4 binding to the XLF promoter. GAPDH was used as a loading control. (C) Silencing of PC4 reduced luciferase activity in both A549 and PC‐9 cells. (D) pCDH‐XLF lentiviral particles were transduced into the above PC4‐silenced NSCLC cells (shPC4 + XLF) to replenish XLF expression. The levels of PC4 and XLF were examined by Western blot. (E) NSCLC cells responses to IR were examined by clonogenic survival assays. Cell resistance to IR was recovered in XLF‐replenishment cells in which PC4 had been depleted (*P <* *0.05, **P <* *0.01, ****P <* *0.0001, Student's t‐test).

To further investigate the effect of PC4 on the expression of XLF, we performed coimmunoprecipitation (Co‐IP) and chromatin immunoprecipitation (CHIP) assays. Co‐IP assays showed that silencing PC4 reduced interaction between PC4 and XLF at the protein level (Fig. 5A). CHIP assays showed that PC4 interacted with the XLF promoter region (Fig. 5B). Subsequently, we used luciferase reporter assays to test whether the repressive function of PC4 inhibition on XLF depends on its binding to the promoter region of XLF. As shown in Fig. 5C, the relative luciferase activity of the reporter was decreased significantly by silencing PC4 (Fig. 5C). Furthermore, when XLF was upregulated in PC4‐knockdown NSCLC cells (Fig. 5D), inhibition of the radioresistance effect was compromised (Fig. 5E). Taken together, these data demonstrated that silencing of PC4 downregulates XLF expression by transcriptionally suppressing XLF, which may be involved in increasing radiosensitivity of NSCLC cells.

### PC4 correlates with the prognosis of patients with NSCLC

**Figure 6:**
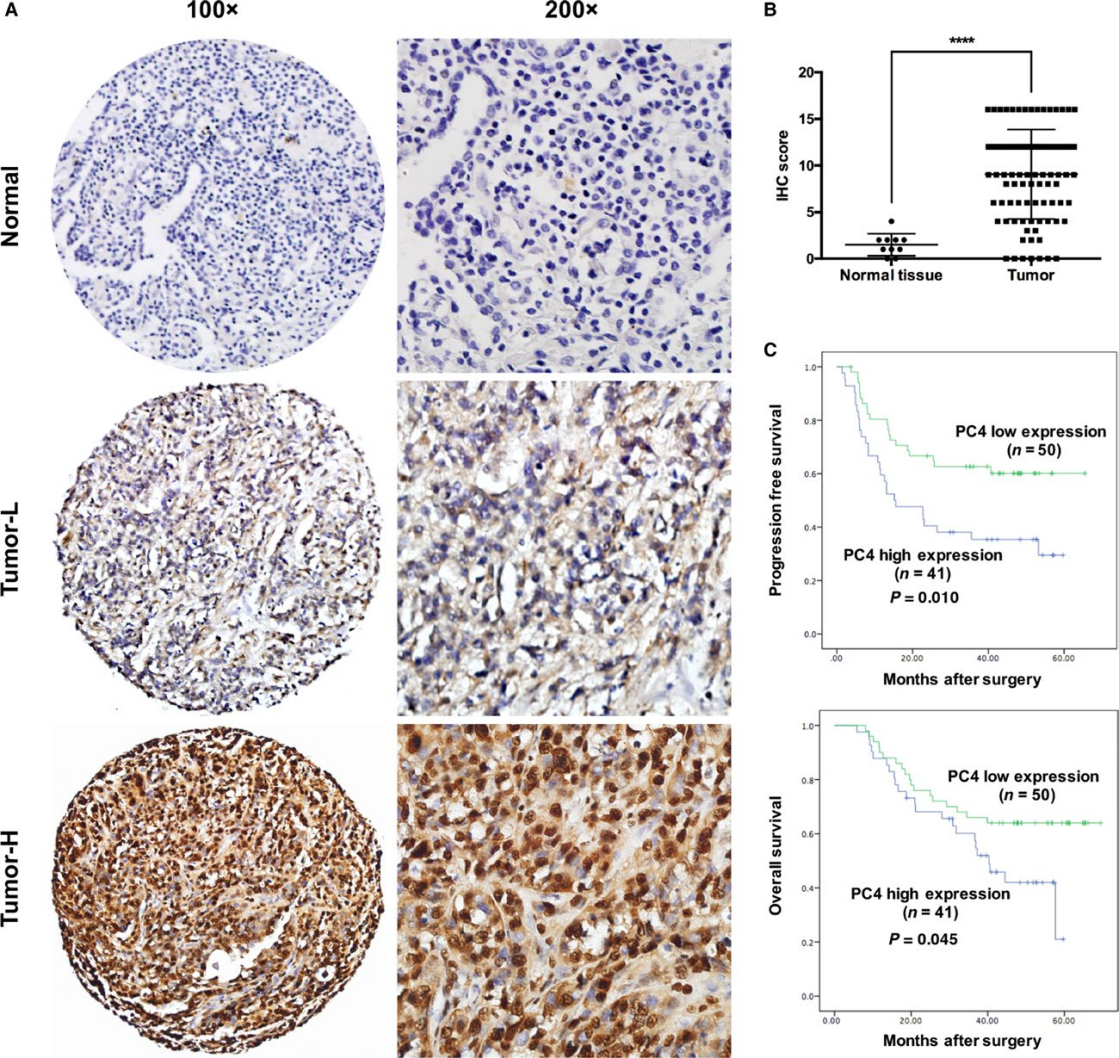
Prognostic significance of PC4 expression in patients with NSCLC. (A) Normal: corresponding normal lung tissue showed low expression of PC4 (IHC score: 0). Tumor‐L: NSCLC tissue exhibited low expression of PC4 (IHC score: 6). Tumor‐H: NSCLC tissue exhibited high expression of PC4 (IHC score: 16). (B) Statistical analysis revealed significantly higher expression of PC4 in NSCLC tissues (*^***^ *P *<* *0.0001, Student's t‐test).(C) Kaplan–Meier plots showed progression‐free survival and overall survival curves of the 91 patients, according to PC4 expression levels in the primary tumor (*P *<* *0.05, log‐rank test).

PC4 expression was examined by immunohistochemistry (IHC) in 91 NSCLCs and in 11 normal lung tissues from Tianjin Medical University Cancer Institute and Hospital. PC4 was upregulated in 45.1% (41 of 91) of NSCLC samples. However, no normal lung samples showed high expression of PC4 (Fig. 6A and B). Correlation analysis demonstrated that high level of PC4 was related to chemoresistance. Moreover, high levels of PC4 was more frequent in chemotherapy noneffective group (SD + PD) than in effective group (PR + CR) (*P *=* *0.028, Table 1).

Most of the patients received chemotherapy or chemoradiotherapy (82 of 91, Table 1). The mean observation period was 38.6 months (5.8–69.5 months). Univariate analysis demonstrated that high PC4 expression was correlated with poor PFS (*P *=* *0.010, Fig. 6C) and OS (*P *=* *0.045, Fig. 6C). Furthermore, PC4 was an independent predictor of PFS (*P *=* *0.044, Table 2).

**Table 2:** Prognostic factors for OS and PFS: univariate and multivariate analyses

Factors	*P* value			
	Univariate		Multivariate	
	OS	PFS	OS	PFS
Pathological lymph node metastasis(pN), pN0‐1 vs. pN2	<0.001^a^	<0.001^a^	0.193	0.284
Pathological stage, I‐II vs. III	<0.001^a^	<0.001^a^	0.275	0.433
Histological type	0.830	0.866		
Histological grade	0.674	0.849		
PC4 expression	0.045^a^	0.01^a^	0.12	0.044^a^

OS, overall survival; PFS, progression‐free survival.

a *P*<0.05

## Discussion

Radiotherapy is widely used to treat NSCLC, and radioresistance is the main reason for failure in cancer treatments. However, the mechanism of NSCLC radiosensitivity is not fully clear. Therefore, it is important to improve the molecular mechanism of NSCLC radiosensitivity in order to improve treatment efficacy.

PC4 was first reported as a transcriptional coactivator in a variety of cellular processes such as DNA damage, replication, and transcription 3. Until now, the mechanism of PC4 in radiosensitivity remains unclear. Our results demonstrated that low expression of PC4 enhanced the radiosensitivity of NSCLC. Apoptosis following unrepaired DNA damages is considered to be the main mechanism of IR‐related cell death 9, 10, 11. We found that knockdown of PC4‐enhanced apoptosis is induced by IR. These results demonstrate that upregulation of PC4 is involved in radioresistance by preventing apoptosis in NSCLC cells.

NHEJ is the main way to repair of DSB 12, 13. In this process, XLF and XRCC4 form helical filaments which may be used to bridge DNA ends for ligation 14, 15, 16. Another study reported that PC4 promotes bridging DNA ends together for ligation, thereby enhancing the effect of DSB repair 6. In our study, after knockdown of PC4, we observed downregulation of XLF, leading to a decrease in XLF foci formation. Downregulation of XLF was accompanied by the increased focus formation of *γ*‐H2AX, which is a feature of DNA damage.

Ahnesorg et al. 17 reported that downregulation of XLF leads to radiosensitivity and impairs NHEJ. Our results showed that XLF was downregulated after PC4 knockdown at both mRNA and protein levels, but the mechanism how XLF is regulated by PC4 remains unclear. In the previous study, PC4 was found to localize on the promoters of neural function‐associated genes 18. Kim et al. 19 found that the N‐terminal helix of SMYD3 and the central *β*‐sheet of PC4 are important for their interaction. Sridharan et al. 20 reported an interaction between the C‐terminal DNA‐binding domain of PC4 and the N‐terminal transactivation domain of p53. Here, our results demonstrate that PC4 directly binds to the XLF promoter region and transcriptionally enhances its expression. Collectively, these results demonstrate that PC4 knockdown radiosensitizes NSCLC cells by transcriptionally regulation of XLF.

Previous studies have shown that PC4 does not influence cell growth under normal physiological condition 3, 21, 22. Therefore, the development of PC4 inhibitors may be a better strategy than other NHEJ inhibitors. Our results demonstrated that PC4 was overexpressed in NSCLC tissues. High expression of PC4 led to chemo‐/radioresistance and was an independent factor for PFS in NSCLC. Therefore, the results suggest that PC4 could be an effective tumor marker to predict the therapeutic response in NSCLC.

In summary, we describe that downregulation of PC4 enhances the radiosensitivity of NSCLC cell both in vivo and in vitro for the first time. In addition, we prove that PC4 knockdown regulates XLF expression by transcriptionally suppressing XLF. Moreover, our results demonstrate that PC4 may be a potential radiosensitivity predictor and an independent prognostic factor for patients with NSCLC.

## Conflicts of Interests

The authors have no conflict of interest.

## Supporting information


**Table S1.** Primers for PCR.
**Table S2.** Apoptosis rate of Mock, shPC4 and shPC4+PC4 group in A549 and PC‐9 cells.

**Data S1.** STR profile report of A549 cell.

**Data S2.** STR profile report of PC‐9 cell.

**Data S3.** WB with molecular weight.

**Data S4.** Supplementary Materials and Methods.
