# Supplementary material for: Inhibition of PC4 radiosensitizes non‐small cell lung cancer by transcriptionally suppressing XLF
Source: Cancer Med. 2018 Mar 9;7(4):1326–37. doi: 10.1002/cam4.1332 (PMC5911594; doi:10.1002/cam4.1332)
Supplement: Supplementary file 1 — Table S1. Primers for PCR. Table S2. Apoptosis rate of Mock, shPC4 and shPC4+PC4 group in A549 and PC‐9 cells. [file CAM4-7-1326-s001.docx]

| **Table S1** Primers for PCR. | | |
| --- | --- | --- |
| Gene | Primer sequence(5'-3') | |
| PC4 | F: | GAGCCCTGTCATCTTCTA |
|  | R: | TTCCTGGTTTCATTTCAC |
| XLF | F: | TCCCAACATTTGATTCGTCCTC |
|  | R: | GCCTTGATGCTTCTGTCCCAC |
| XLF promoter | F: | TGCTGCTAAACATCCTACACTG |
|  | R: | TCCAGGGAGAAAAGGCCAG |
| GAPDH | F: | GTTCGACAGTCAGCCGCATCT |
|  | R: | 5ʹ-CCTGCAAATGAGCCCCAGCCT-3ʹ |

**Table S2** Apoptosis rate of Mock, shPC4 and shPC4+PC4 group in A549 and PC-9 cells.

|  |  | Mock | | | shPC4 | | | shPC4+PC4 | | |
| --- | --- | --- | --- | --- | --- | --- | --- | --- | --- | --- |
| A549 | 0Gy | 1.0% | 4.7% | 2.5% | 1.4% | 4.1% | 2.9% | 1.1% | 2.2% | 2.5% |
|  | 6Gy | 8.7% | 11.7% | 13.3% | 23.1% | 19.0% | 30.4% | 11.4% | 13.7% | 15.8% |
| PC-9 | 0Gy | 2.5% | 4.7% | 2.5% | 2.9% | 4.1% | 2.7% | 2.2% | 4.8% | 2.8% |
|  | 6Gy | 11.7% | 11.0% | 12.3% | 17.0% | 23.1% | 18.1% | 13.7% | 14.2% | 13.2% |
