# Supplementary material for: Inhibition of PC4 radiosensitizes non‐small cell lung cancer by transcriptionally suppressing XLF
Source: Cancer Med. 2018 Mar 9;7(4):1326–37. doi: 10.1002/cam4.1332 (PMC5911594; doi:10.1002/cam4.1332)
Supplement: Supplementary file 3 — Data S2. STR profile report of PC‐9 cell. [file CAM4-7-1326-s003.pdf]

GENETIC TESTING BIOTECHNOLOGY

Cell Line Authentication Service

STR Profile Report

Sample Submitted By: Dr. Ping Wang
Tianjin Medical University Cancer Institute and Hospital
Email Address: wangping@tjmuch.com
Sales Order: 170408A
Cell Line Designation: PC-9
Date Sample Received: Apr 8th, 2017
Report Date: Apr 10th, 2017

Methodology: Nineteen short tandem repeat (STR) loci plus the gender determining locus, Amelogenin, were amplified using the commercially available EX20 Kit from AGCU. The cell line sample was processed using the ABI Prism® 3500 Genetic Analyzer. Data were analyzed using GeneMapper® ID-X v1.4 software (Applied Biosystems). Appropriate positive and negative controls were run and confirmed for each sample submitted.

Data Interpretation: Cell lines were authenticated using Short Tandem Repeat (STR) analysis as described in 2012 in ANSI Standard (ASN-0002) by the ATCC Standards Development Organization (SDO) and in Capes-Davis et al., Match criteria for human cell line authentication: Where do we draw the line? Int J Cancer. 2013;132(11):2510-9.

GTB™ performs STR Profiling following ISO 9001:2008 and ISO/IEC 17025:2005 quality standards.
There are no warranties with respect to the services or results supplied, express or implied, including, without limitation, any implied warranty of merchantability or fitness for a particular purpose. Genetic Testing Biotechnology (GTB) is not liable for any damages or injuries resulting from receipt and/or improper, inappropriate, negligent or other wrongful use of the test results supplied, and/or from misidentification, misrepresentation, or lack of accuracy of those results. Your exclusive remedy against GTB and those supplying materials used in the services for any losses or damage of any kind whatsoever, whether in contract, tort, or otherwise, shall be, at GTB's option, refund of the fee paid for such service or repeat of the service.

The GTB™ is a registered trademark of Genetic Testing Biotechnology Corporation (Suzhou).

Technical Questions?
GTB Technical Support
+86-512-62806339
STR_service@163.com
Section 303, Yixin BLD
SIP, Suzhou, 215123
Jiangsu, P.R. China

Ordering Questions?
STR_order@163.com
GTB Corporation
+86-512-62806339
Section 303, Yixin BLD
SIP, Suzhou, 215123
Jiangsu, P.R. China

GENETIC TESTING BIOTECHNOLOGY

Cell Line Authentication Service

STR Profile Report

Sales Order: 170408A

Test Results for Submitted Sample			DSMZ Reference Database Profile	
**Loci**	**Query Profile:** PC-9		**Database Profile:** PC-9	
Amelogenin	X		X	
D3S1358	16			
D13S317	8		8	
D7S820	10	11	10	11
D16S539	9		9	
Penta E	11	15		
TPOX	11		11	
TH01	7		7	
D2S1338	19	20		
CSF1PO	11		11	
Penta D	9	13		
D19S433	13	15.2		
vWA	17		17	
D21S11	29	30		
D18S51	15			
D6S1043	13	19		
D8S1179	11	15		
D5S818	11		11	
D12S391	18			
FGA	23			

The allele match algorithm compares the 8 core loci plus amelogenin only, even though alleles from all loci will be reported when available.

Note: Loci highlighted in grey (8 core STR loci plus Amelogenin) can be made public to verify cell identity. In order to protect the identity of the donor, ***please do not publish*** *the allele calls from all the STR loci tested.*

Explanation of Test Results

Cell lines with ≥80% match are considered to be related; i.e., derived from a common ancestry. Cell lines with between a 55% to 80% match require further profiling for authentication of relatedness.

- ☐ The submitted sample profile is human, but not a match for any profile in the DSMZ STR database.
- ☑ The submitted profile is an exact match for the following human cell line(s) in the DSMZ STR database (8 core loci plus Amelogenin): PC-9
- ☐ The submitted profile is similar to the following DSMZ human cell line(s):

e-Signature, Technician:

数字签名者：Faye Wong
DN：cn=Faye Wong, o=Genetic Testing Biotechnology Corporation (Suzhou), ou=DNA Typing Section, email=str_service@163.com, c=CN
日期：2017.04.10 15:34:23 +08'00'

e-Signature, Reviewer:

数字签名者：Alan Cui
DN：cn=Alan Cui, o=Genetic Testing Biotechnology Corporation (Suzhou), ou=Supervision Section, email=str_order@163.com, c=CN
日期：2017.04.10 15:35:53 +08'00'

More information

Addendum: Electropherogram for the customer’s sample set 1 of 1

GENETIC TESTING BIOTECHNOLOGY

Cell Line Authentication Service

STR Profile Report

Applied Biosystems

GeneMapper® ID-X 1.4

Project: 170410

Sample Name	Panel	SQO

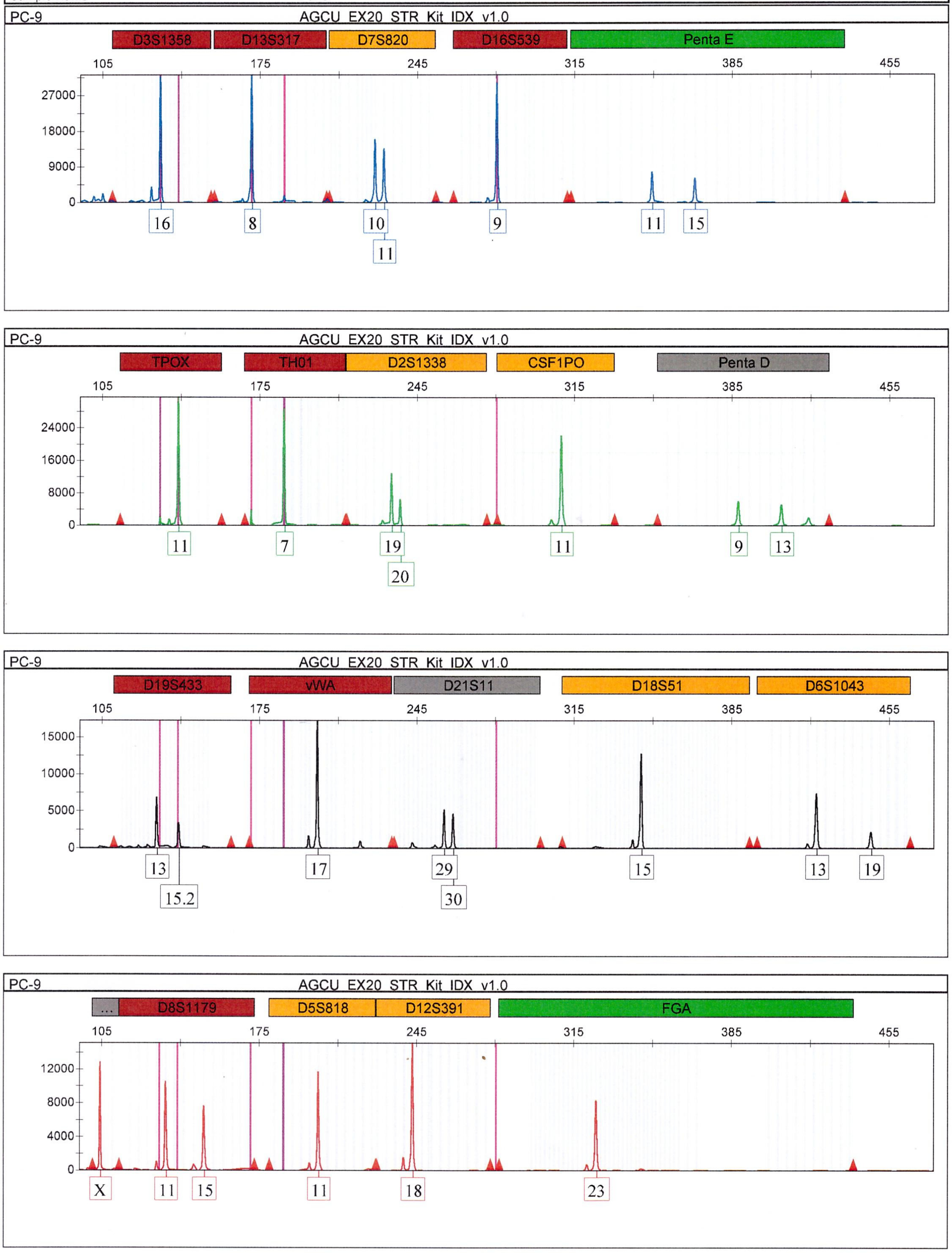

星期一 四月 10,2017 02:42下午, CST   Printed by: Administrator   Page 1 of 1
