# Supplementary material for: Inhibition of PC4 radiosensitizes non‐small cell lung cancer by transcriptionally suppressing XLF
Source: Cancer Med. 2018 Mar 9;7(4):1326–37. doi: 10.1002/cam4.1332 (PMC5911594; doi:10.1002/cam4.1332)
Supplement: Supplementary file 4 — Data S3. WB with molecular weight. [file CAM4-7-1326-s004.pdf]

Figure 1

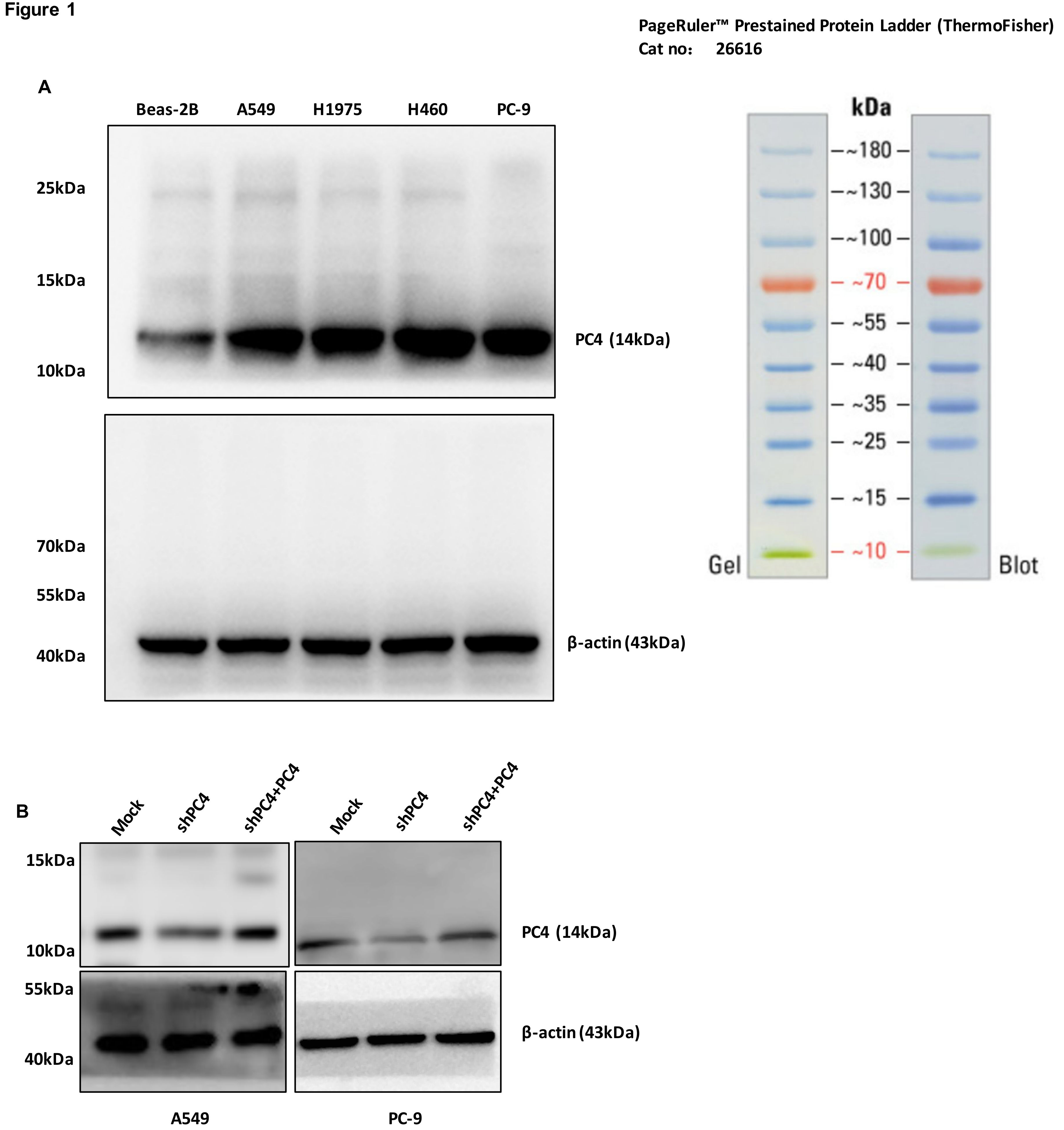

PageRuler™ Prestained Protein Ladder (ThermoFisher)
Cat no: 26616
A
Beas-2B
A549
H1975
H460
PC-9
25kDa
15kDa
10kDa
PC4 (14kDa)
70kDa
55kDa
40kDa
β-actin (43kDa)
kDa
~180
~130
~100
~70
~55
~40
~35
~25
~15
~10
Gel
Blot
B
Mock
shPC4
shPC4+PC4
Mock
shPC4
shPC4+PC4
15kDa
10kDa
PC4 (14kDa)
55kDa
40kDa
β-actin (43kDa)
A549
PC-9

Figure 2

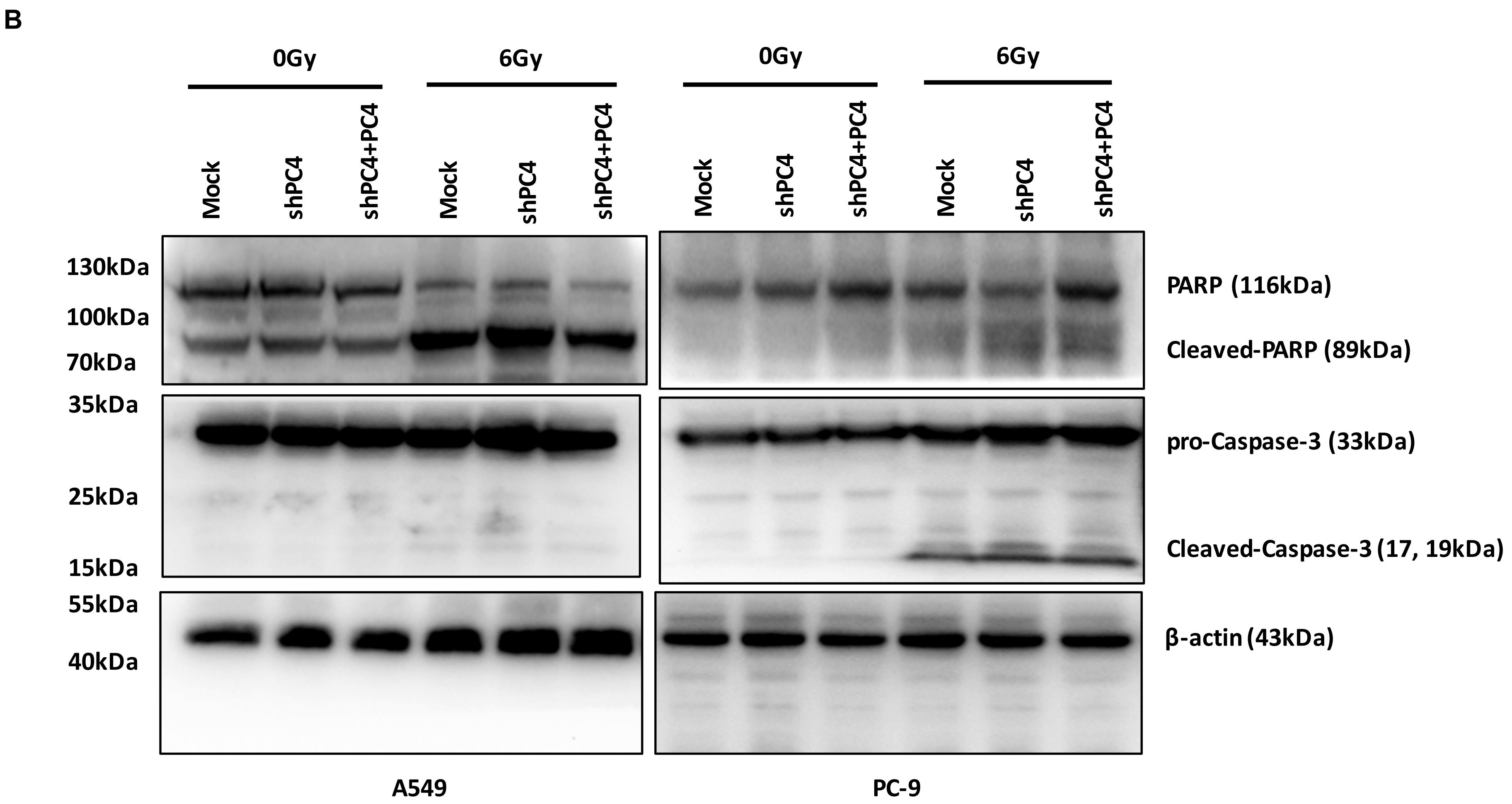
B
0Gy
6Gy
0Gy
6Gy
Mock
shPC4
shPC4+PC4
130kDa
100kDa
70kDa
35kDa
25kDa
15kDa
55kDa
40kDa
PARP (116kDa)
Cleaved-PARP (89kDa)
pro-Caspase-3 (33kDa)
Cleaved-Caspase-3 (17, 19kDa)
β-actin (43kDa)
A549
PC-9

Figure 4

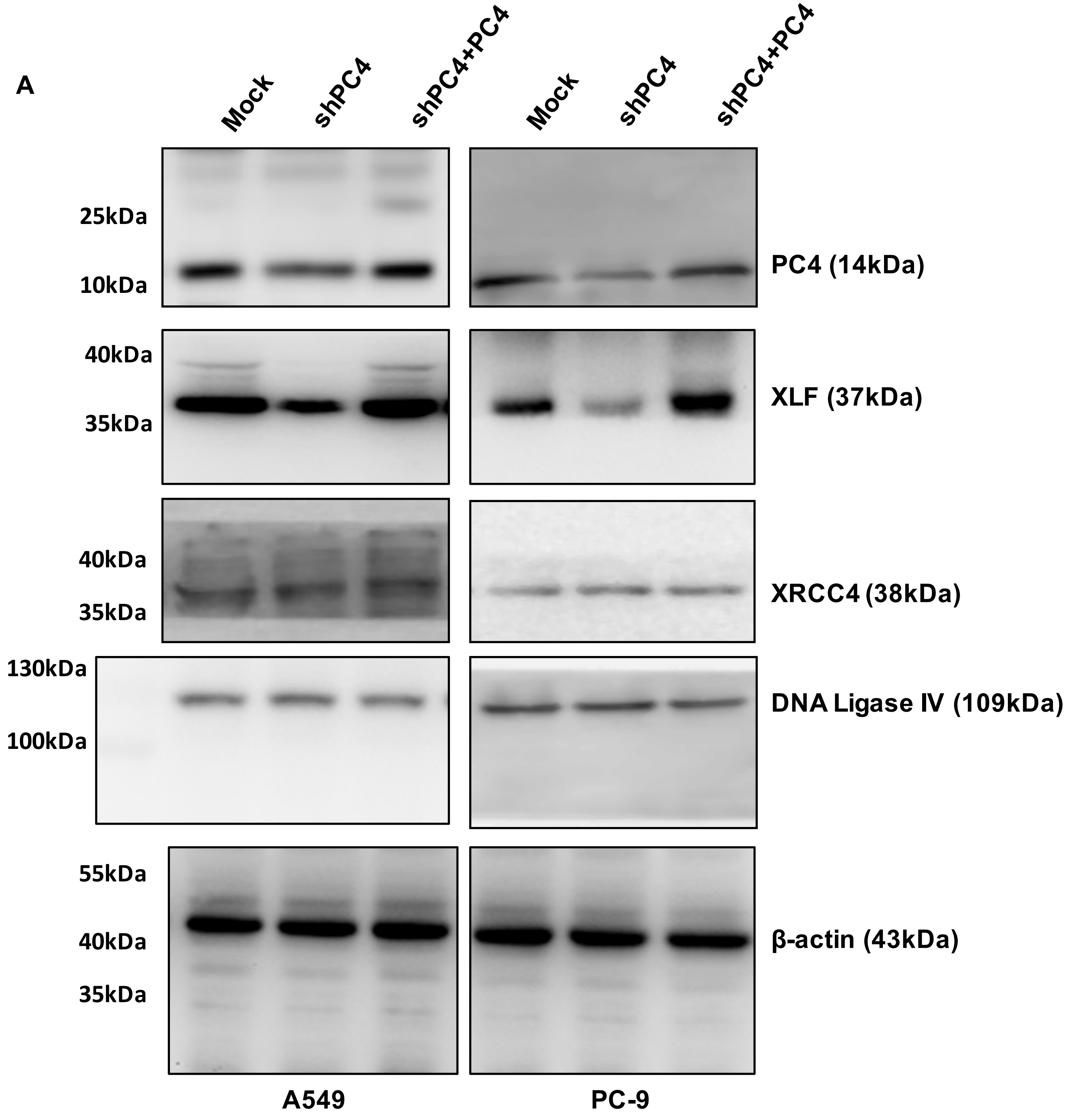
A
Mock
shPC4
shPC4+PC4
Mock
shPC4
shPC4+PC4
25kDa
10kDa
PC4 (14kDa)
40kDa
35kDa
XLF (37kDa)
40kDa
35kDa
XRCC4 (38kDa)
130kDa
100kDa
DNA Ligase IV (109kDa)
55kDa
40kDa
35kDa
β-actin (43kDa)
A549
PC-9

Figure 5 A

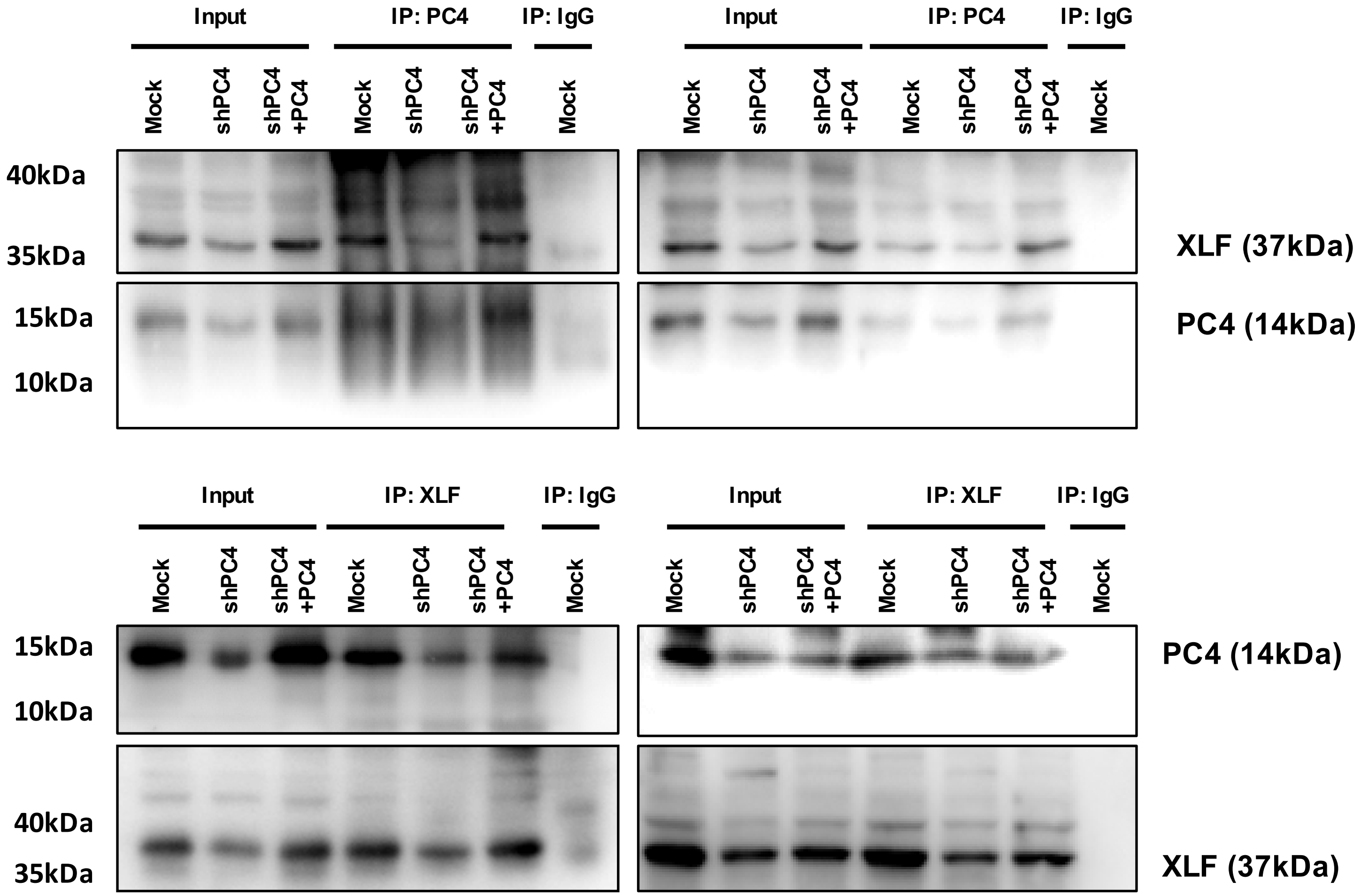

Input
IP: PC4
IP: IgG
Mock
shPC4
shPC4 +PC4
40kDa
35kDa
15kDa
10kDa
XLF (37kDa)
PC4 (14kDa)
IP: XLF

Figure 5

D

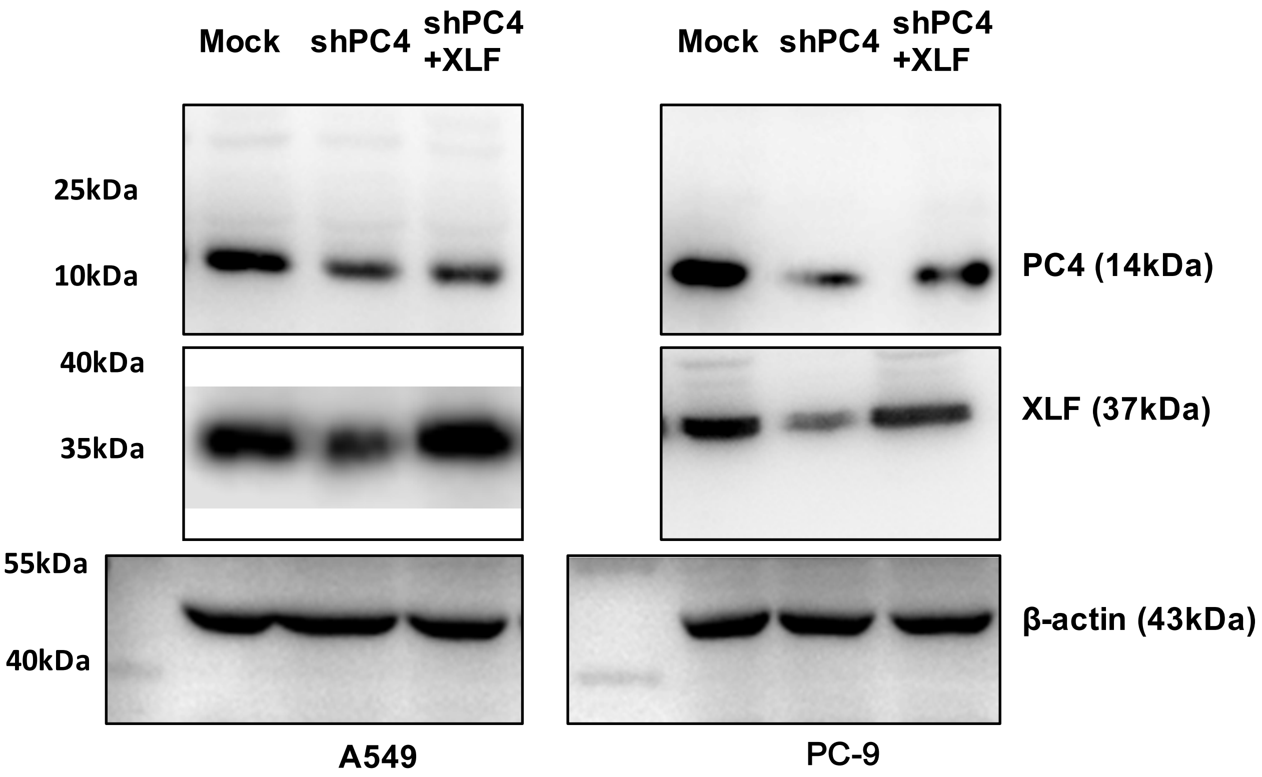
Mock
shPC4
shPC4 +XLF
Mock
shPC4
shPC4 +XLF
25kDa
10kDa
40kDa
35kDa
55kDa
40kDa
PC4 (14kDa)
XLF (37kDa)
β-actin (43kDa)
A549
PC-9
