# Supplementary material for: Inhibition of PC4 radiosensitizes non‐small cell lung cancer by transcriptionally suppressing XLF
Source: Cancer Med. 2018 Mar 9;7(4):1326–37. doi: 10.1002/cam4.1332 (PMC5911594; doi:10.1002/cam4.1332)
Supplement: Supplementary file 5 — Data S4. Supplementary Materials and Methods. [file CAM4-7-1326-s005.docx]

**Supplementary Materials and Methods**

**Co-immunoprecipitation (Co-IP).** Cell extracts were prepared with the RIPA buffer. 200 μg Proteins were incubated at 4°C overnight with a rabbit anti-XLF, a mouse anti-PC4 antibody, or a control IgG. The immune complexes were immuno-precipitated on Protein A/G Plus-Agarose beads (Santa Cruz), washed for 3 times with TBST, resolved on 10% SDS gel, and detected with anti-XLF antibody.

**ChIP assays.** ChIP assays were performed using PC4 and XLF antibodies (CST). A549 and PC-9 cells were crosslinked with 1% formaldehyde for 15 min at room temperature. Formaldehyde was quenched by adding glycine (final concentration, 0.125 M). After washing the cells with cold PBS solution, we collected the cells with lysis buffer (0.5% NP-40, 25 mM HEPES, 150 mM KCl, 1.5 mM MgCl_2_, 10% glycerol and KOH (pH 7.5)) containing proteinase inhibitors and further incubated the cells on ice for 15 min. Cell lysates were centrifuged (5,000 rpm for 5 min), and supernatants were discarded. Cell lysates were subjected to sonication with ChIP-radioimmunoprecipitation assay lysis buffer (50 mM Tris, pH 8.0; 150 mM NaCl; 0.1% SDS, 0.5% deoxycholate, 1% NP-40 and 1 mM EDTA; 10 times, 30 s on/30 s off) and were centrifuged (13,200 rpm for 30 min). Supernatant from lysates was immunoprecipitated with antibody overnight at 4 °C and was pulled down using protein A/G PLUS-Agarose (Santa Cruz) by centrifugation (3,400 rpm for 2 min). Immuno-precipitates were further washed serially with ChIP-radioimmunoprecipitation assay, high salt (50 mM Tris, pH 8.0; 500 mM NaCl; 0.1% SDS, 0.5% deoxycholate, 1% NP-40 and 1 mM EDTA), LiCl wash buffer (50 mM Tris, pH 8.0; 1 mM EDTA, 250 mM LiCl; 1% NP-40 and 0.5% deoxycholate) and Tris-EDTA buffer. Finally, immunoprecipitate crosslinking was reversed by incubation at 65 °C overnight, and immunoprecipitates were treated with RNase A and proteinase K to extract DNA. ChIP amplicons were detected via ChIP-PCR. Primer sequence for XLF promoter is showed in Reverse transcription-PCR.
